# Effects of reminiscence therapy on quality of life and life satisfaction of the elderly in the community: a systematic review

**DOI:** 10.1186/s12877-023-04001-1

**Published:** 2023-07-10

**Authors:** Eunyoung Shin, Myeongshin Kim, Seyoon Kim, Sohyune Sok

**Affiliations:** 1 Department of Nursing, Kyung Bok University, Gyeonggi, Republic of Korea; 2 Department of Nursing, Graduate School, Kyung Hee University, Seoul, Republic of Korea; 3 College of Nursing Science, Kyung Hee University, 26, Kyungheedae-ro, Dongdaemun-gu, Seoul, 02447 Republic of Korea

**Keywords:** Aged, Personal satisfaction, Reminiscence, Quality of life

## Abstract

**Background:**

Reminiscence therapy is known as an effective intervention method for elderly with various health conditions. This study was to provide basic data for contributing to the spread and development of effective intervention by analyzing the characteristics and effects of reminiscence therapy applied to the elderly at home.

**Methods:**

To select the article to be studied, literature published from January 2000 to January 2021 was searched through eight databases. A total of 897 articles were searched, and the collected papers were analyzed using the flow chart of Preferred Reporting Items for Systematic Review and Meta-Analysis (PRISMA). Of these, 6 articles suitable for the selection criteria were selected by reviewing titles and abstracts, excluding duplicate papers, using EndNote X9 and Excel 2013. The quality of literature was evaluated using the critical appraisal checklist of the Joanna Briggs Institute.

**Results:**

For the characteristics of the selected literature, most of the literature published within the last 10 years was conducted, and the research design was conducted only in experimental research. The most common form of reminiscence therapy was group reminiscence, and the type was ‘simple reminiscence’. The intervention method of reminiscence therapy was provided in various ways, but ‘Sharing’ was mainly used, and the representative topic of recall was ‘Hometown’. Most of the intervention was performed less than 10 times and took about 60 min.

**Conclusion:**

According to the results of this study, reminiscence therapy provided to the elderly living in the community was effective in improving their quality of life and life satisfaction. Therefore, it is suggested that reminiscence therapy can be an intervention method that is helpful for positive psychological factors and health promotion by improving the quality of life and life satisfaction of the elderly living in the community, and further, it is considered that the elderly can contribute to non-pharmacologically healthy aging in the community.

## Introduction

The proportion of the elderly population aged 65 years and older in South Korea was 15.7% in 2020. Based on this information, it is predicted to reach 20.3% in 2025, with Korea becoming a super-aged society [1]. Moreover, the life expectancy of Koreans would be 82.7 years, exceeding the average of 80.7 years in major OECD (organization for economic co-operation and development) countries, just a gap of 1.5 years with Japan’s 84.2 years, the country with the longest life expectancy [2]. Such a rapid increase in the elderly population may overload medical facilities and welfare facilities in relation to physical and mental health problems [3]. Furthermore, the elderly health care system was problematic in terms of the lack of elderly health care providers and inconsistent and insufficient physical and mental health services [4]. As a result, it is necessary to review the national and regional policies on health care services for the elderly population. In particular, it is necessary to shift from the high cost and unsustainable approach to an efficient and preventive care method in relation to the current health care-related services, including a paradigm shift towards a prevention-oriented, community-based approach to health care for the elderly [5].

Reminiscence therapy is known as an effective intervention method for elderly subjects with various health conditions, from dementia [6] to long-term care [7], hospice [8], and the elderly without cognitive problems [9]. The main characteristic of reminiscence therapy is to help them recall past events, emotions, thoughts, etc., increase positive psychological factors, such as quality of life and life satisfaction, and adapt well to the present situation [10]. Moreover, reminiscence therapy can be implemented in various environments (community residence, nursing home, hospital, etc.) of elderly subjects, and can be provided by a trained expert in a group or individual format [11]. Reminiscence therapy is relatively easy to implement compared to other interventions, and it is cost-effective with minimal side effects [12]. Reminiscence therapy can be classified into three categories according to the type of therapeutic task: 1) simple reminiscence is to recall and share memories and stories selected by individuals with the goal of improving happiness, self-esteem, and social interaction of the elderly in a short period of time, 2) life review is to integrate negative and positive memories by dealing with the whole life story in accordance with the goal of supporting the elderly with mild levels of stress, and 3) life review therapy is to reminisce the past, the present, and the future together, and integrate the individual’s memories with the goal of relieving stress for the elderly who experience severe stress, such as depression or anxiety [13].

Life satisfaction, which is evaluated as the most important positive psychological factor among the elderly, is an index of physical and mental health [14], and is one of the variables that is highly correlated with their quality of life [15]. Although the quality of life and the life satisfaction of the elderly are lower than those of other age groups [16], various fields and communities are initiating efforts to improve them [17]. A recent study reported that reminiscence therapy improved the quality of life and life satisfaction of the dementia elderly [6, 18]. However, although reminiscence therapy was effective in maintaining and alleviating the depression or psychosocial health of the elderly living in the community [19], published literature reviews that provide an integrated and appropriate overview for improving the quality of life deeply related to the successful aging of the elderly are scarce [20, 21]. Therefore, this study was conducted to analyze the characteristics and the effects of reminiscence therapy applied to the elderly at home, thereby contributing to the spread and development of effective interventions and providing fundamental data for improving the quality of life and life satisfaction, one of the positive psychological factors of the elderly at home.

The purpose of this study was to conduct a systematic review based on the results of experimental research that applied the reminiscence therapy to improve the quality of life and life satisfaction of the elderly living in the community in order to verify the effectiveness and the validity of reminiscence therapy.

## Methods

This study was a systematic literature review conducted in accordance with research guidelines provided by the Cochrane Handbook for Systematic Reviews of Interventions and the PICO framework (Population, Intervention, Comparison, and Outcome).

### Study design

This study is a systematic review of experimental research to integrate and analyze the effects of reminiscence therapy on the quality of life and life satisfaction of the elderly living in the community.

### Study selection criteria

To select studies, key questions were selected to understand the effect of reminiscence therapy applied to the elderly living in the community on quality of life and life satisfaction, and then an electronic database search was conducted according to specific inclusion and exclusion criteria.

### Inclusion criteria

The inclusion criteria for the specific data search are as follows: (1) The study subjects were the elderly living in the community, and the elderly diagnosed with dementia was excluded. (2) The selected study intervention was reminiscence therapy, and included group reminiscence, individual reminiscence, simple reminiscence, life review, and life review therapy. (3) The comparison target was the group that did not receive the experimental treatment and intervention for reminiscence therapy, or received alternative intervention. (4) As for the intervention outcome, quantitative values ​​that can analyze life satisfaction and quality of life applied with reminiscence therapy were selected. (5) As for the study type, RCT study and non-RCT study were selected.

### Exclusion criteria

For data analysis, (1) studies for the elderly subjects admitted to nursing homes or hospitals, not the elderly living in the community, (2) experimental studies conducted with a single arm and qualitative studies, (3) studies and dissertations published only in abstracts, and (4) studies not published in Korean or English were excluded.

### Literature search strategy

#### Search database

A literature search was conducted systematically on journals published from January 2000 to January 2021 using an electronic database. Since the 2000s, the increase in the global elderly population and related research have been actively conducted, and based on this, the search period was selected. Research Information Service System (RISS), Korean Studies Information Service System (KISS), National Discovery for Science Library (NDSL), DBpia, Korean Medical Database (KMbase) were used for domestic literature searches. PubMed, Ovid MEDLINE, Cochrane Library, Cumulative Index to Nursing and Allied Health Literature (CINAHL) were used to search foreign literature

### Search terms

For the Korean article search, (‘elderly’ or ‘elderly at home’ or ‘community’), (‘reminiscence’ or ‘reminiscence therapy’ or ‘reminiscence therapy’ or ‘non-drug’), and (‘well-being’ or ‘satisfaction with life’ or ‘satisfaction’ or ‘quality of life’) were used, and the search terms for foreign articles was (“Elderly” [MeSH] or “Older Adult” [MeSH] or “Aged” [MeSH] or community dwelling or community dwelling elderly or community dwelling older), (“Reminiscence” [MeSH] or “Reminiscence Therapy” [MeSH] or reminiscence program or reminiscence intervention or spiritual reminiscence or memory reminiscence or life review or life history), and (Well-being or Life Satisfaction or Satisfaction or “Quality of Life” [MeSH] or QoL or Health Related QoL).

### Literature selection process

After searching the literature through the search terms presented in the database, in order to select the articles that will be analyzed, the title and the abstract were first reviewed in stages, and the inclusion and exclusion criteria were applied. If it was difficult to select the articles based on the abstract alone, the full text of the article was found and reviewed to check whether it met the inclusion criteria, and whether it was duplicated.

**Fig. 1:**
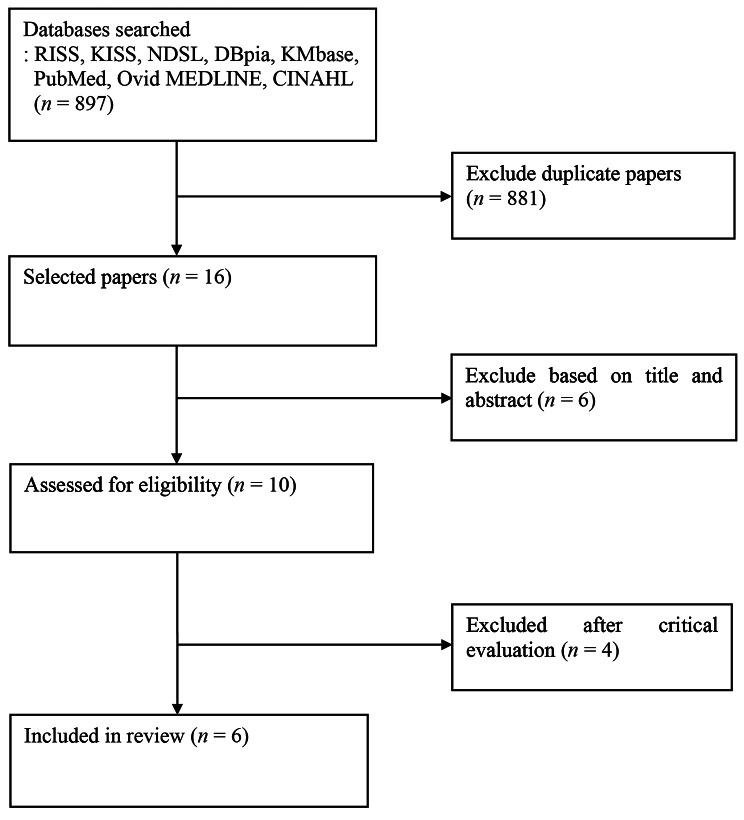
Flow diagram for study selection

A total of 138 articles were searched for Korean articles primarily through the database, and 759 articles were searched for foreign articles, leading to a total of 897 articles. Duplicate papers were identified using EndNote X9 and Excel 2013, and 16 papers were selected excluding 881 papers. After reviewing the titles and abstracts of the selected articles, 10 articles that met the selection criteria were selected. After checking the original texts of these papers, 4 papers that did not correspond to the key questions were excluded, and therefore, the final 6 domestic and foreign papers were selected. (Fig. 1). The selection process of all the articles was independently performed by two researchers, and the final articles were decided through a discussion with all the researchers in case of disagreement, and during the professional review stage of the final articles.

### Assessment of literature quality

To objectively and comprehensively assess the literature selected in this study, the quality assessment tool of the Joanna Briggs Institute (JBI) was used. Based on the design of the literature, the 13-item JBI Critical Appraisal Checklist for Randomized Controlled Trials was used for the RCT study, and the 9-item JBI Critical Appraisal Checklist for Quasi-Experimental Studies was used for the non-RCT study. Each item is composed to respond to one of Yes, No, Unclear, or Not Applicable, and it is designed to review the adequacy of the assessment items among researchers, not to assess the quality of the literature on a quantitative basis. However, in order to improve methodological rigor, and to assess biases and threats to efficacy, five or more items should be rated Yes. Therefore, to evaluate the quality of the literature in this study, two researchers conducted it independently, and each researcher scored 5 or more Yes on the two checklists. In addition, the literature was reviewed until a point of agreement was reached through discussion. Through the review, evaluation, and discussion process, 4 articles that did not meet the inclusion criteria were excluded, and finally, 6 articles were included after confirmation by two researchers.

### Data analysis

To abstract all data of the finally selected articles, the characteristics of the variables were systematically organized by using the coding table prepared by the researchers. The coding table was composed of publication year, theoretical framework, research design, study population, intervention, measurement instrument, and dependent variables etc. To maintain the accuracy of the data analysis, two researchers independently evaluated the accuracy of data analysis, and through a consensus, the results were integrated, and descriptive analysis was performed.

### Ethical consideration

This study was conducted after obtaining approval from the K University Institutional Review Board (IRB No. KHSIRB-20-193 [RA]).

## Results

### General characteristics and research methodology

Among the experimental studies that verified the effect of reminiscence therapy on the quality of life and life satisfaction of the elderly subjects living in the community from January 2000 to January 2021, a total of 6 articles were included in the systematic review of this study. As for publication year, 4 articles (66.7%) were published within the last 10 years from 2011 to 2021, and 2 articles (33.3%) were published from 2000 to 2010. As for the use of the theoretical framework, 5 articles (83.3%) did not use it, and only 1 article (16.7%) was used. As for the type of study design, 5 articles (83.3%) were non-RCT studies, and 1 article (16.7%) was an RCT study. As for the study’s sample size, 3 articles (50%) had less than 30 subjects, 2 articles (33.3%) had 30 or more and 49 or fewer subjects, and 1 article (16.7%) had 50 or more subjects. The mean age of the subjects was 70 years or older in 4 articles (66.7%), and 60 years or older and 69 years or less than 69 years in 2 articles (33.3%). Elderly people have differences in their physical health, socio-psychological status, and activities of daily living depending on their age group (e.g., early elderly, middle elderly, and late elderly), and these may affect reminiscence therapy [22]. Therefore, it was analyzed by dividing the average age based on 70 years old (Table 1).

**Table 1:** General characteristics and research methodology

Variables		Category	*n* (%)
General characteristics	Publication year	2001–2010 2011–2021	2 (33.3%) 4 (66.7%)
	Use of theoretical framework	Used Not used	1 (16.7%) 5 (83.3%)
Research methodology	Type of study	Non-RCT RCT	5 (83.3%) 1 (16.7%)
	Total sample size (n)	< 30 30–49 ≥ 50	3 (50%) 2 (33.3%) 1 (16.7%)
	Mean age of participants (year)	60–69 ≥ 70	2 (33.3%) 4 (66.7%)

### Method of application of reminiscence therapy

As for the format in which reminiscence therapy was provided, group reminiscence was in 5 articles (83.3%), and individual reminiscence was in 1 article (16.7%). As for the number of interventions, 4 articles (66.7%) applied single intervention, and 2 articles (33.3%) applied multiple intervention. As for the type of reminiscence therapy, simple reminiscence was in 4 articles (66.7%), life review was in 2 articles (33.3%). And as for the number of times of intervention, 4 articles (66.7%) had less than 10 times, and 2 articles (33.3%) had 10 or more times. As for the time per session, 3 articles (50%) had less than 60 minutes, and 3 articles (50%) had 60 or more than 60 minutes (Table 2).

**Table 2:** Method of application of reminiscence therapy

Variables	Category	*n* (%)
Intervention unit	Group reminiscence Individual reminiscence	5 (83,3%) 1 (16.7%)
The number of interventions	Single intervention Multiple intervention	4 (66.7%) 2 (33.3%)
Reminiscence type	Simple reminiscence Life review reminiscence	4 (66.7%) 2 (33.3%)
The number of times	< 10 10 ≤	4 (66.7%) 2 (33.3%)
Time per session (min)	< 60 60 ≤	3 (50%) 3 (50%)

### Assessment and results of quality of life and life satisfaction

As for the outcome variables of the study that applied reminiscence therapy to the elderly living in the community, 2 articles (33.3%) had quality of life and 4 articles (66.7%) had life satisfaction. As for the instrument used in measuring quality of life, 1 article each (50%) used the Oxford Happiness Questionnaire and the Short Form-36 Quality of Life for quality of life, respectively. The instrument used in measuring life satisfaction, 2 articles (50%) used the Life Satisfaction Scale for Elderly, 1 article (25%) used the Life Satisfaction Index-A, and 1 article (25%) used the Philadelphia Geriatric Center Morale Scale (Table 3).

**Table 3:** Assessment and results of quality of life and life satisfaction

Variables	*n* (%)	Measurement tool	*n* (%)
Quality of life	2 (33.3%)	Short Form-36 Quality of Life Oxford Happiness Questionnaire	1(50%) 1(50%)
Life satisfaction	4 (66.7%)	Life satisfaction scale for elderly Life satisfaction index-A Philadelphia geriatric center morale scale	2(50%) 1(25%) 1(25%)

### Comparison of the effects of reminiscence therapy

Looking at the main results according to the type of reminiscence therapy, the dependent variables that showed a significant effect in the 4 articles that provided simple reminiscence were quality of life (1 article) and life satisfaction (3 articles), as well as depression (2 out of 4 articles), psychological well-being (2 out of 4 articles), and self-esteem (1 out of 4 articles). Also, in 2 studies that provided life review, a significant effect was shown in quality of life (1 article) and life satisfaction (1 article), respectively, as well as depression (1 out of 2 articles), physiological indicators (1 out of 2 articles), self-esteem (1 out of 2 articles), and cognitive function (1 out of 2 articles) (Table 4).

**Table 4:** Comparison of the effects of reminiscence therapy (N = 6)

Authors (year)	Theoretical framework	Research design	Intervention				Dependent variables (Study results)
			Type	Contents	Methods	No. of times & Length (min)	
Cho (2001)	Cognitive, psychosocial development, and life review theory	Non-equivalent control group pre-posttest design	Group & Simple	Family and home, Disease and treatment, Religion	Sharing memories with others	4 times & 60 min	Life satisfaction (*), Self-esteem
Lee, Cho, Yoon, & Son (2008)	N/A	Non-equivalent control group pre-posttest design	Group & Life review	Hometown, Motivating of health, Family future plan	Sharing memories with others and Horticultural therapy	18 times & 120 min	Life satisfaction (*), Self-esteem (*), Depression (*), Physiological indicators (*)
Meléndez-Moral, Charco-Ruiz, Mayordomo-Rodríguez, & Sales-Galán (2013)	N/A	Non-equivalent control group pre-posttest design	Group & Simple	Hometown	Sharing memories with others	8 times & 60 min	Life satisfaction (*), Self-esteem (*), Depression (*), Psychological well- being (*)
Sok (2015)	N/A	Non-equivalent control group pre-posttest design	Individual & Life review	Hometown, Folk remedy, Philosophy and religion	Viewing pictures	4 times & 60 min	Quality of life (*), Cognitive function (*)
Yousefi, Sharifi, Tagharrobi, & Akbari (2015)	N/A	RCT	Group & Simple	All about personal life	Sharing memories with others	6 times & 120 min	Life satisfaction (*)
Viguer, Satorres, Fortuna, & Meléndez (2017)	N/A	Non-equivalent control group pre-posttest design	Group & Simple	All about an individual’s daily life	Sharing memories with others	10 times & 120 min	Depressed mood (*), Life satisfaction (*), Psychological well- being (*)

* *P*-value < 0.05

The results of this study showed that the intervention effects according to the type of reminiscence therapy varied, such as physical and psychological state, as well as improvement of the quality of life and life satisfaction of the elderly at home.

## Discussion

This study intended to systematically review the literature on the effects of reminiscence therapy on quality of life and life satisfaction, one of the positive psychological factors of the elderly living in the community, in order to confirm the effects of interventions, and compare the characteristics of various approaches and corresponding results. A total of 6 articles were selected for this study, and the study method of each study, the characteristics of the subjects, and the characteristics of the interventions were analyzed. The 6 articles selected in this study were found to be much smaller than the results of systematic reviews on other topics. Because reminiscence therapy is an effective intervention to enhance their socio-psychological factors, mainly targeting the elderly with dementia [6, 22], it is relatively infrequently applied to the normal elderly without dementia. In addition, reminiscence therapy is mainly an intervention provided by trained professionals [11], and the intervention is applied to the elderly living or registered in elderly-related institutions. Accordingly, the accessibility of reminiscence therapy is relatively low for the elderly living in the community. However, in a super-aged society, health care for the elderly should be done with a prevention-centered, community-based approach, including the elderly in the community [5], and this suggests that studies evaluating the application and effectiveness of reminiscence therapy should be actively conducted on them.

As a result, it was found that there were more articles published within the last 10 years than those published from 2000 to 2010 among all articles. This is consistent with the results of the research that interest has recently been increasing in improving the health and living standards of the elderly in the community [5]. In addition, it is considered to be a study result suggesting the importance of positive psychological factors such as quality of life and life satisfaction as well as health management of not only the elderly with dementia or the elderly in facilities but also the normal elderly in the community are expanding. In the study design type, it was found that non-RCT studies were more frequent than RCT studies, and reminiscence therapy was effective in quality of life and life satisfaction in non-RCT as well as RCT studies. The results of non-RCT studies may have lower representativeness or validity than those of RCT, but it is thought that 5 non-RCT studies additionally performed repeated measurements, post-survey, or increased sample size to compensate for the limitations. Studies using a theoretical framework were found to be fewer than those without. In Cho [23]’s study using a theoretical framework, the program was constructed based on cognitive theory, developmental stage theory, and recall theory that can explain the characteristics of the elderly in order to apply group reminiscence therapy. Cho [23] selected the topic of reminiscence therapy through cognitive theory, constructed the intervention process through developmental stage theory, and set the application period of intervention through recall theory. As a result, the group reminiscence therapy developed based on the three theories was effective in improving the life satisfaction of the elderly, which is considered to be a systematic approach to the recall response, one of the psychological adaptation processes in old age. Therefore, reminiscence therapy to which a theoretical framework is applied can be an intervention that can better reflect the psychological problems of old age than reminiscence therapy without a theoretical framework [23, 24]. As for the sample size of the study, 3 articles had less than 30 subjects and 3 articles had 30 or more subjects. So, it is considered that studies applying parametric statistics with a sample size of 30 or more must be conducted to increase the reliability of study results in the future. Then the mean age of the subjects was 70 years or older in most articles, which is consistent with the results of the studies on the deterioration of the quality of life according to the increase in the age of the elderly [16].

The results of this study showed that the number of applications of reminiscence therapy and the application time per intervention vary for each previous study. The number of applications of reminiscence therapy varied from a minimum of 4 times to a maximum of 18 times. In particular, 4 times was the most common with 2 studies. In previous studies, In previous studies, it was reported that the number of applications of recall therapy that was effective for the life satisfaction of the elderly in the community was 8 times [25], but the results of this study showed that the number of applications of reminiscence therapy was not sufficient. Among the study results in which reminiscence therapy was applied 4 times, it was not effective for self-esteem, especially during group reminiscence. The self-esteem of the elderly is one of the most important concepts for improving their quality of life [26]. Therefore, it is thought that 12 or more times of group reminiscence therapy should be provided for the elderly in the community to improve their self-esteem and quality of life. The application time per session of reminiscence therapy varied from a minimum of 60 minutes to a maximum of 120 minutes. In particular, 60 minutes was the most common with 3 articles. These study results are consistent with the previous study results in which 60 minutes is appropriate as the application time of reminiscence therapy to improve the quality of life and life satisfaction of the elderly [27].

The results of this study showed that group reminiscence therapy was mainly used as the method of application of reminiscence therapy, which is consistent with the study results of group reminiscence therapy being more effective than individual reminiscence therapy in improving the negative psychological factors of the elderly [7]. Also, it was found that simple reminiscence was mainly used among the types of reminiscence therapy, which is considered to be an appropriate reminiscence therapy in improving the positive psychological factors of the elderly living in the community with relatively stable mental health [13]. Moreover, as the intervention method of reminiscence therapy, all studies used sharing, and the selected reminiscence topic was family. These results are consistent with the results of previous studies [28–31] that they were the most effective intervention method, and the topic of reminiscence therapy improved the negative psychological factors of the elderly.

The study results showed that measurement instruments used to assess the quality of life of the elderly living in the community were the Oxford Happiness Questionnaire (OHQ) and the Short Form-36 Quality of Life (SF-36 QoL). OHQ is an indicator of whether an individual is performing well in social functions, and is a tool that can reasonably evaluate the quality of life [32], and SF-36 QoL is a tool that measures the quality of life related to health [33]. Although these tools were not developed considering the characteristics of the quality of life of the elderly, they have the characteristic that they can be used at any time by a human being. In the results of this study, both tools demonstrated high validity and reliability in measuring the quality of life, and reminiscence therapy was found to be effective in improving the quality of life of the elderly at home. However, since these tools measure only the psychological factors of the subjects according to the items, they are very limited in reflecting the characteristics of the elderly and evaluating their overall quality of life. Therefore, it is considered that it is necessary to actively use tools to measure the quality of life of the elderly, such as the Geriatric Quality of Life scale, and to evaluate their characteristics reflecting their characteristics. Moreover, in the results of this study, the most frequently used measurement instrument to evaluate life satisfaction was the Life Satisfaction Scale for Elderly, which had high reliability and validity in measuring the life satisfaction of the elderly at home [34]. Thus, it is considered that Life Satisfaction Scale for Elderly will be very effective when evaluating the effect of reminiscence therapy on the life satisfaction of the elderly.

Considering the previous studies that revealed that positive psychological factors, such as quality of life and life satisfaction, are correlated with cognitive function as well as physical and psychological state, this study suggests that, in the long term, reminiscence therapy can serve as a cornerstone for preventing dementia of the elderly living in community, and managing their physical/psychological health to improve and maintain their quality of life and life satisfaction. Therefore, it was confirmed that reminiscence therapy is a very effective intervention method not only for the elderly with cognitive function problems or dementia, but also for the elderly at home. In the future, it is necessary to develop and apply a program that utilizes reminiscence therapy to improve the quality of life and life satisfaction among the elderly living in the community.

### Limitations

In study limitations, this study selected documents published in Korean or English and performed data analysis, and there is a limitation in generalizing the results of this study because documents written in other languages were not included. In addition, since two researchers reviewed the literature during the selection process, there may be differences with the research results derived from multiple researchers, so there is a possibility that there may be a bias in the research results. However, this study helps utilize reminiscence therapy for improving quality of life and life satisfaction of the elderly living in the community and is particularly helpful for health professionals who are active in the community. And, as the result of this study was applied to the normal elderly, it is not recommended for the elderly with psychiatric problems, dementia, or living in institutions in the future.

## Conclusions

Reminiscence therapy provided to the elderly living in the community improved their quality of life and life satisfaction. In particular, group reminiscence therapy was found to be more effective than individual reminiscence therapy. Moreover, reminiscence therapy was effective in improving depression, physical/mental health, and cognitive function of the elderly at home. However, it is necessary to consider the individual characteristics of the elderly during group reminiscence therapy, and additional research is needed on the effect of reminiscence therapy on self-integration in the elderly. Therefore, it suggests that reminiscence therapy can be an intervention method that is helpful in positive psychological factors and health promotion, not only for the elderly with cognitive function problems. Furthermore, reminiscence therapy will be able to contribute to the elderly’s healthy aging in the community without the administration of drugs.

## Data Availability

The datasets generated and/or analyzed during the current study are not publicly available due no new data were created or analyzed in this study, but are available from the corresponding author on reasonable request.
